# A rapid and cost-effective fluorescence detection in tube (FDIT) method to analyze protein phosphorylation

**DOI:** 10.1186/s13007-016-0143-5

**Published:** 2016-11-03

**Authors:** Xiao Jin, Jin-Ying Gou

**Affiliations:** State Key Laboratory of Genetic Engineering, Institute of Plant Biology, Collaborative Innovation Center for Genetics and Development, School of Life Sciences, Fudan University, Shanghai, 200438 China

**Keywords:** Protein kinase reaction, Fluorescence, Pro-Q^®^ diamond, Wheat Kinase START 1, Thylakoid ascorbate peroxidase

## Abstract

**Background:**

Protein phosphorylation is one of the most important post-translational modifications catalyzed by protein kinases in living organisms. The advance of genome sequencing provided the information of protein kinase families in many organisms, including both model and non-model plants. The development of proteomics technologies also enabled scientists to efficiently reveal a large number of protein phosphorylations of an organism. However, kinases and phosphorylation targets are still to be connected to illustrate the complicated network in life.

**Results:**

Here we adapted Pro-Q^®^ Diamond (Pro-Q^®^ Diamond Phosphoprotein Gel Stain), a widely used phosphoprotein gel-staining fluorescence dye, to establish a rapid, economical and non-radioactive fluorescence detection in tube (FDIT) method to analyze phosphorylated proteins. Taking advantages of high sensitivity and specificity of Pro-Q^®^ diamond, the FDIT method is also demonstrated to be rapid and reliable, with a suitable linear range for in vitro protein phosphorylation. A significant and satisfactory protein kinase reaction was detected as fast as 15 min from Wheat Kinase START 1.1 (WKS1.1) on a thylakoid ascorbate peroxidase (tAPX), an established phosphorylation target in our earlier study.

**Conclusion:**

The FDIT method saves up to 95% of the dye consumed in a gel staining method. The FDIT method is remarkably quick, highly reproducible, unambiguous and capable to be scaled up to dozens of samples. The FDIT method could serve as a simple and sensitive alternative procedure to determine protein kinase reactions with zero radiation exposure, as a supplementation to other widely used radioactive and in-gel assays.

## Background

During evolution, living organisms developed accurate and strict cellular regulatory systems to control all biological aspects in a cell from gene transcription to protein post-translational modifications (PTMs). Protein phosphorylation is the most abundant and widespread PTM, and represents over 50% of PTMs, although more than 300 different types of PTMs have been reported (http://www.abrf.org). Tightly regulated and rapid reversible protein phosphorylation affords organisms the advantages to survive, via adding or removing a phosphoryl group to targets catalyzed by protein kinases or phosphatases through consuming ATP, the most commonly used cofactor in life, which saves both time and energy [1]. At the protein level, phosphorylation is critical for the structural and functional state of proteins to function properly, e.g. stability, subcellular-localization, biochemical activity and degradation [2]. At the cellular level, protein phosphorylation is essential in almost all biological processes, including DNA replication, gene transduction, cell cycle progression, cell differentiation, cell metabolism, and signaling [3].

In plants, protein phosphorylation is predominant in regulations of both normal development and responses to diseases caused by pathogenic bacteria or fungi, as revealed by previous intensive studies of plant scientists [4–6]. Poplar 5-hydroxyconiferaldehyde O-methyltransferase 2 (PtrAldOMT2), a central enzyme in monolignol biosynthesis, was reduced by 60% in its specific biochemical activity upon phosphorylation, an on/off switch in this context [7]. In wheat, a strip rust resistance gene Wheat Kinase START 1 (WKS1.1 for the full length protein, thereafter) phosphorylates and inhibits a thylakoid ascorbate peroxidase (tAPX, thereafter), leading to accumulation of H_2_O_2_ and cell death [8]. Phosphorylations in the above enzymes are included in the term of phosphoproteome, referring to all phosphorylated proteins or peptides in a living organism.

Phosphoproteome studies have developed significantly due to technological advances, such as sample preparation and phosphoprotein (peptide) enrichment, MS fragmentation and sensitivity, and also advances in phosphoproteome databases and bioinformatics for phosphoprotein prediction and annotation [9–12]. In *Arabidopsis*, 29,058 phosphorylation sites in 9003 phosphoproteins have been reported to cover 40% of the Arabidopsis genome, which indicates that protein phosphorylation is a fundamental PTM in plants [13]. This is consistent with the fact that there are 1052 protein kinases in *Arabidopsis* genome, accounting to 4.8% of the whole genome, compared with 518 (0.5–2.5%) in human genome [14, 15]. Therefore, the information of protein kinases is almost complete in a sequenced genome, and the information of proteins phosphorylated in the organism is also more and more abundant due to technological break-throughs in the fast growing phosphoproteomics field [16, 17]. However, only ∼6.6% of protein phosphorylations have been linked to particular kinases in open protein phosphorylation databases [10]. And even for those predictable kinase-phosphorylation networks, biochemical experimental approaches to connect protein kinases and protein phosphorylations are still indispensible to reveal biological significances of the phosphorylation events [18].

There are several widely used assays to analyze protein phosphorylation, based on radioactive ATP, gel retardation, phospho-antibody, and fluorescence staining respectively [8]. The radioactive ATP method use [γ-^32^P] ATP in a kinase reaction to transfer the ^32^P group to a target protein, which was subsequently quantified by the radioactivity to monitor the phosphorylation process [19]. This method affords a high sensitivity but ^32^P is a radioactive hazard, which makes it unsuitable for high throughput assays and unfriendly to the environment [20]. A retardation gel contains some chemicals, e.g. Phospho-tag, to bind and slow down the phosphorylated proteins specifically during electrophoresis which were then recognized by a specific antibody in Western Blot to show the phosphorylation status [21]. The cost of Phospho-tag is high and a specific antibody against the target protein is needed. In the phospho-antibody method, phosphorylated protein was recognized specifically by antibodies generated against different phosphorylated protein, peptides, or amino acids, e.g. anti-phosphotyrosine antibodies [22]. However, low affinity and specificity limited the application of antibodies against phospho-Ser/Thr sites, which account for 77.99 and 17.81% of total phosphorylations in *Arabidopsis* phosphoproteomics, respectively [23]. The phosphorylated protein specific antibodies are not as widely available in plants as in animal studies, which is partially due to their high-cost and long time to develop. In the fluorescence staining method, the phosphorylated protein was separated in SDS-PAGE gel and stained by Pro-Q^®^ Diamond (Pro-Q), a widely used dye to stain phosphorylated proteins with high sensitivity and specificity [24]. This method consumed 60 ml of the expensive dye to stain one gel, and is not suitable to analyze a large number of samples at the same time.

**Fig. 1:**
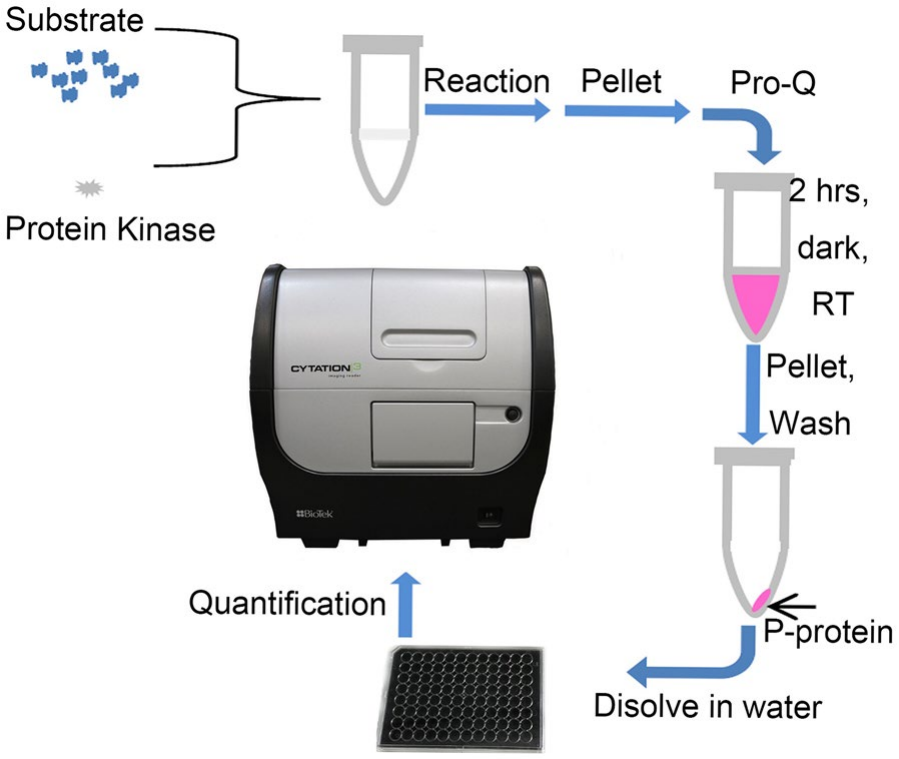
Schematic representation of the workflow used for the fluorescence detection in tube (FDIT) method. The protein kinase reaction products in a tube were pelleted by cold acetone, stained in Pro-Q^®^ Diamond for 2 h in dark, then pelleted again in cold acetone, washed to remove the stain residues and quantified with a plate reader. *P*-*protein* phosphorylated protein

In this study, we adapted the laborious Pro-Q^®^ Diamond gel staining method to establish a fluorescence detection in tube (FDIT) method. To overcome the disadvantage of high cost of Pro-Q^®^ Diamond (60 ml per gel) in the gel staining method, we stained the phosphorylated protein in a 1.5 ml tube with only 0.1 ml of Pro-Q^®^ Diamond and optimized the conditions for both staining and washing. After being stained in a tube, the protein was pelleted by cold acetone, washed to remove the dye residue, air-dried and dissolved in water. The fluorescence signal in the sample was subsequently quantified with a plate reader (Fig. 1). This FDIT method is demonstrated as a rapid, cost-effective method, and is easy to be scaled up for protein kinase reactions, while it still keeps the advantage of high specificity and sensitivity of Pro-Q^®^ Diamond. The FDIT method was successfully applied to determine the kinase reaction of a wheat kinase (WKS1.1) and its protein target (tAPX), and revealed a significant and satisfactory protein kinase reaction. This FDIT method can be widely applied in biochemical studies of plant protein kinases.

## Methods

### Protein in tube staining procedure

Casein (Sangon Biotech (Shanghai) Co., Shanghai, China) were homogenized and suspended in Mili-Q water at the concentration of 0.2 µg per µl. For the staining duration time analysis, 10 µl of casein was mixed with 100 µl of Pro-Q^®^ Diamond (Molecular Probes, Life Technologies Corporation, Grand Island, NY, USA) and kept in dark at RT for 10, 20, 30, 60 and 120 min. Then the protein was pelleted with 10 volumes of cold acetone (Sinopharm Chemical Regent Co. Ltd Shanghai, China) kept in a −20 °C freezer, and centrifuged at 13,200 rpm for 1 h at 4 °C. Supernatant was carefully drained out and discarded without touching the protein pellet. The pellet was rinsed with 0.5 ml of cold acetone and centrifuge to remove the supernatant twice. The pellet was air-dried and dissolved in 200 µl of Mili-Q water and moved to a black 96 well plate (Costor, Corning, NY USA). Fluorescence signal at 590 nm (excited at 530 nm) were measured in a Cytation3 microplate reader (Biotek, Winooski, VT, USA). To analyze the washing effect, the sample was stained for 2 h and the pellet was washed for 0, 1, 2 and 3 times with cold acetone and quantified as described above. To build the standard curve, different amounts of casein, ovalbumin (OVA) (Sigma-Aldrich Chemical Co., St. Louis, MO, USA) and BSA (Sinopharm Chemical Regent Co. Ltd Shanghai, China) were stained for 2 h and washed 3 times before quantified as described above. All samples were analyzed with four biological repeats. Background fluorescence signals (15,500, 19,100 and 14,600) were removed from each sample. Data analyses were processed in Microsoft Excel.

### Protein in gel staining

Different amount of OVA and BSA were first separated in a similar gel by SDS PAGE. One gel was stained with Coomassie brilliant blue R250 solution in (50% ethanol and 10% acetic acid in water), distained with distaining buffer (50% ethanol and 10% acetic acid in water) and photographed in G:BOX XR5 Gel imaging system (Syngene, Cambridge, UK). Pro-Q^®^ Diamond gel staining was done according to the user manual. In brief, the gel was fixed in 50% methanol plus 10% acetic acid for 30 min twice, washed 3 times with ultrapure water each for 10 min, stained with 60 ml Pro-Q^®^ Diamond stain for 90 min, distained with 20% acetonitrile in 50 mM sodium acetate pH4.0, wash twice with ultrapure water and imaged with Typhoon FLA 9000 (GE Healthcare Waukesha, WI, US).

### Protein kinase reaction and staining

tAPX expression and purification were described in an early study [8]. The full-length cDNA of WKS1 was inserted in pET41b and were transformed into BL21 (DE3) plysS competent cells (Promega, Madison, WI USA). Bacteria were grown in LB medium to OD_600_ 0.6–0.8 first at 37 °C and then reduced to 18 °C before induction with 0.5 mM IPTG overnight. The cells were collected and suspended in 1/10 volume of protein lysis buffer (100 mM Tris–HCl pH8, 500 mM NaCl, 1 mM β-mercaptoethanol, 20 mM imidazole, 10% glycerol, 0.1% Triton X-100) with Complete Mini Protease Inhibitor Cocktail Tablets (Roche Diagnostics Corporation, Indianapolis, IN USA). The cells were sonicated three times each for 30 s and incubated for 1 h with shaking in ice. After centrifugation at 13,200 rpm for 1 h, the supernatant was removed to a new tube, and mixed with 0.5 ml pre-equilibrated Ni–NTA resin (Pierce Biotechnology Inc., Rockford, IL USA) for 1 h in ice. The samples were then loaded into columns and washed with 20 volumes of washing buffer (100 mM Tris–HCl pH8.0, 500 mM NaCl, 1 mM β-mercaptoethanol, 20 mM imidazole, 10% glycerol). Recombinant protein was mixed with 1 ml of Elution Buffer (100 mM Tris–HCl pH8.0, 500 mM NaCl, 1 mM ascorbic acid, 250 mM imidazole, 10% glycerol), held for 10 min, and then eluted. The protein was dialyzed overnight against dialysis buffer (100 mM HEPES pH 7.4, 500 mM NaCl, 1 mM ascorbic acid, 10% glycerol). Protein concentration was quantified with BCA Protein Quantification Kits (Yeasen, Shanghai, China).

For kinase reaction time analysis, 2 µg of tAPX was mixed 0.2 µg of WKS1.1 in a kinase reaction buffer (100 mM PBS pH 7.5, 10 mM MgCl_2_, 1 mM ascorbic acid) at room temperature for 15, 30, 60, 90 and 120 min before pellet with 10 folds of cold acetone. The product was stained as described in the standard curve part. For kinase amount analysis, 2 µg of tAPX was mixed 0.1, 0.2, 0.5, 1.0 and 2.0 µg of recombinant WKS1.1 protein in a kinase reaction buffer for 15 min, 10 folds of cold acetone was added to stop the kinase reaction and stained as described before.

Four biological repeats were analyzed for each sample and the data was analyzed in Microsoft Excel.

## Results and discussion

### Optimal conditions for the FDIT method

**Fig. 2:**
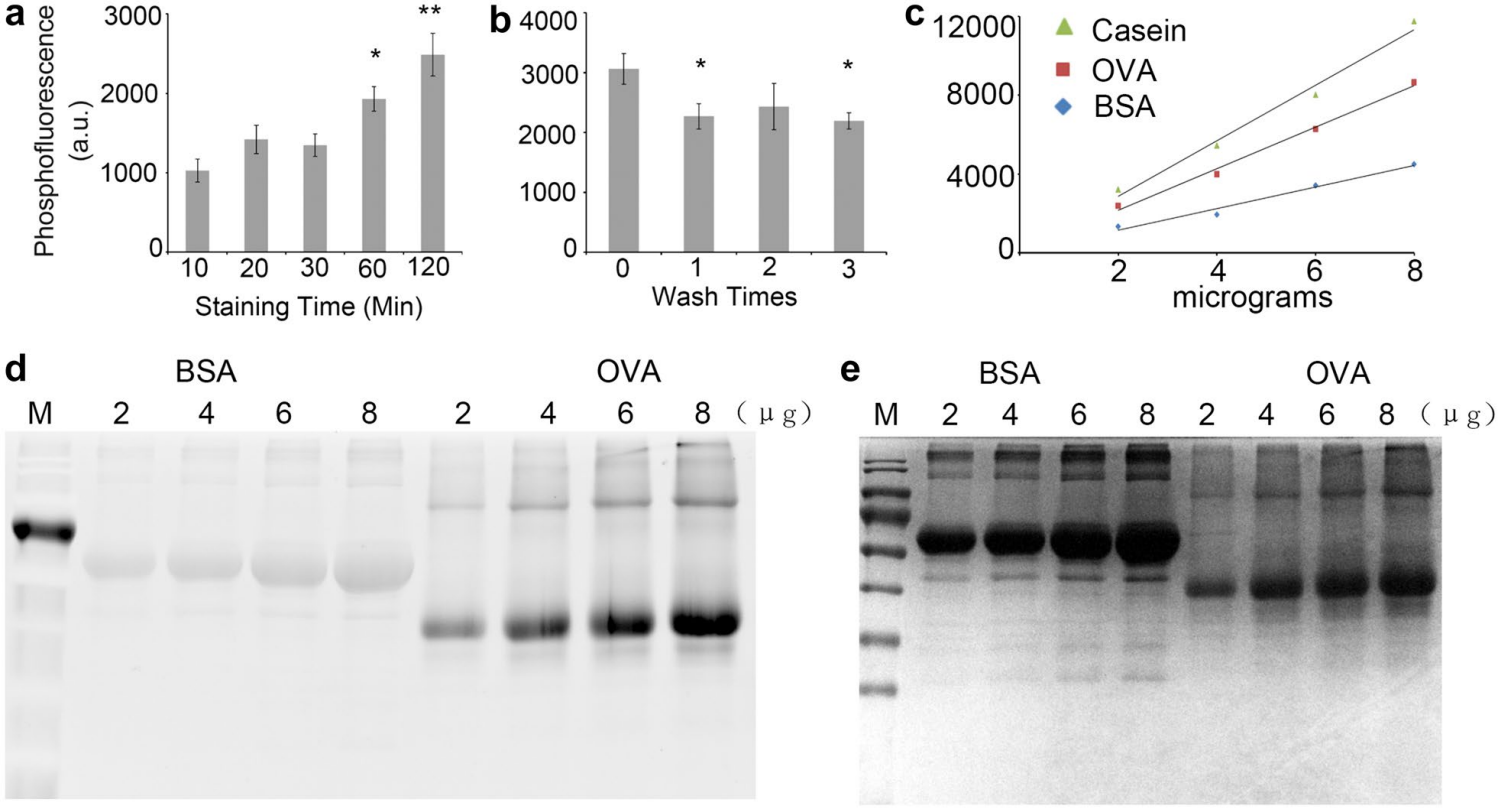
Determination of the optimum conditions for the FDIT method. **a** Effect of staining duration time on the fluorescence signal. Significant changes were found between 60/120 (min) and other samples (student’s *t* test *p* value <0.05), but not in between 60 and 120 (min) samples (student’s *t* test *p* value = 0.06). **Student’s *t* test *p* value <0.01, *student’s *t* test *p* value <0.05 compared with samples of 10–30 min. **b** Effect of washing times on the fluorescence signal. Significant changes were found between unwashed and washed samples, but not in washed samples with different washing times. *Student’s *t* test *p* value <0.05 compared with the sample without wash. n = 4. *Bars* stand for ± standard error. **c** Linear dynamic regression range of FDIT method. Phospho-fluorescence signals from different amounts of casein, OVA and BSA were collected and plotted with the mean of 4 biological repeats. **d**, **e** Pro-Q^®^ Diamond and Coomassie brilliant blue R250 staining of OVA and BSA in the linear range. M stands for protein size marker and the number shows micrograms of samples loaded

To reduce the cost of phosphoprotein staining, we performed an in-tube staining of casein, a widely used phosphoprotein marker, with only 0.1 ml of Pro-Q^®^ Diamond, a specific and sensitive phosphoprotein dye [24]. First, we analyzed the effect of staining duration time on the phosphoprotein fluorescence signal. A significant increase (student’s *t* test *p* value <0.05) of fluorescence signal was detected in the samples stained for 1 h compared with those stained shorter, from 10 to 30 min (Fig. 2a). A further increase was detected in the samples stained for 2 h, but the change was not significant (student’s *t* test *p* value = 0.06) (Fig. 2a). Thus, we concluded that a staining period of 1–2 h was sufficient to give an optimal signal.

To determine the effect of washing, we quantified the fluorescence signals from the samples with different washing times. The protein was pelleted in 10 volumes of cold acetone, which dissolved the Pro-Q^®^ Diamond very well. The majority of the free dye in the solution was removed during the pellet step, as shown by the significant decrease in the fluorescence of those washed samples (Fig. 2b). No difference was detected between samples with one and two or more washing steps, indicating that 3 washes can sufficiently reduce the noise and that the phosphor-fluorescence signal in the pellet was stable in the washing process (Fig. 2b).

Pro-Q^®^ Diamond dye displayed a beautiful linear range from 0.1 to 1 μg phosphoprotein per band in the gel staining method (MAN0002351, Molecular Probes, Inc). In a typical kinase reaction, however, 2–4 μg of substrate was used in one reaction [25]. We analyzed the linear range of FDIT method in a higher concentration (2–8 μg) with casein, OVA and BSA, representing high, low or no phosphorylation standards (MAN0002351, Molecular Probes, Inc). Over the tested range (2–8 μg), the fluorescence signal was proportional to the protein amount. Interestingly, satisfactory linear correlations were detected between the phosphofluorescence signal and protein amount from 2 to 8 μg with R^2^ over 0.95 (Fig. 2c). The casein standard curve had the highest slope while that of OVA sit in the middle and the BSA one was the lowest (Fig. 2c). This finding is consistent with the fact that casein has 5–8 phosphorylations per molecule, while OVA has 2 and BSA has no phosphorylation (MAN0002351, Molecular Probes, Inc). Thus the detection range of FDIT method could satisfy the requirement of typical protein kinase reactions in vitro.

To compare the in tube staining method and in gel staining method, 2–8 µg of BAS and OVA were separated by SDS PAGE gel and stained with Coomassie brilliant blue R250 to show the total protein and Pro-Q^®^ Diamond stain to show the phosphorylated protein. OVA samples have much stronger staining than BSA (Fig. 2c). However, a nonspecific staining was observed by Pro-Q^®^ Diamond stain in the BSA sample which was predicted to contain no phosphorylation group. This is consistent with the result of weak stain in gel on a non-phosphorylated protein, lysozyme in the user manual.

### The application of the FDIT method in a protein kinase reaction

To test the application of FDIT method in a real protein kinase reaction, we studied the kinase reaction of Wheat Kinase START 1 (WKS1.1) on a thylakoid ascorbate peroxidase (tAPX), which was established in our earlier work [8]. We conducted a kinase reaction using 0.2 μg of WKS1.1 with 2 μg of tAPX, which were both purified from prokaryotic expression recombinant proteins, respectively. After reaction, the samples were first pelleted with cold acetone and then stained with 100 μl of Pro-Q^®^ Diamond, pelleted with 10 folds of cold acetone, washed twice with cold acetone, air-dried, dissolved in 200 μl of water and quantified with a plate reader.

**Fig. 3:**
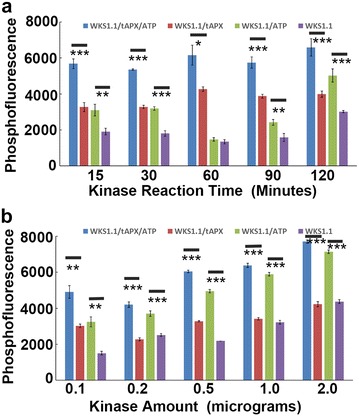
Application of the FDIT method in a kinase reaction. **a** Effect of reaction time. ATP was added to initiate auto-phosphorylation (WKS1.1) or the kinase reaction (WKS1.1 + tAPX) of WKS1.1 and the reactions were stopped at different times. Samples without ATP serve as negative controls. **b** Effect of the kinase amount. ***Student’s *t* test *p* value <0.001, **student’s *t* test *p* value <0.01, *student’s *t* test *p* value <0.05. n = 4. *Bars* stand for ± standard error

A very significant increased fluorescence signal was found in the sample with kinase reaction upon ATP initiation (Student’s *t* test *p* value <0.01) (Fig. 3a). Interestingly, a significant increase was also detected in the sample with WKS1.1 alone with ATP, indicating a self-phosphorylation of WKS1.1, which was consistent with our early study [9]. However, the fluorescence signal of tAPX phosphorylation was significantly stronger than auto-phosphorylation (Student’s *t* test *p* value <0.05), and this increase was produced by the phosphorylation on tAPX in the WKS1.1-tAPX kinase reaction (Fig. 3a).

Longer kinase reactions were also conducted to monitor the kinase reaction process, in the samples of tAPX with WKS1.1 no significant difference was detected in the samples reacted for 15–120 min indicating that 15–30 min are sufficient for WKS1.1 kinase reaction, even though longer reactions had slightly higher signals (Fig. 3a). To test the kinase reaction sensitivity, different amounts of kinase were added into the solution, higher signals could be detected in the samples with high content of WKS1.1 (Student’s *t* test *p* value <0.05), but these changes were mainly contributed by the auto-phosphorylation of WKS1.1 (Fig. 3b). Therefore, a low kinase/substrate ratio is preferred in the FDIT method if the kinase has an auto-phosphorylation activity.

It is noticeable that in the samples without ATP, a phosphor-fluorescence signal increase was also detected when tAPX was added (tAPX/WKS1.1) compared with WKS1.1 alone (Fig. 3a, b). This was predicted to arise from the non-specific binding of tAPX with the Pro-Q^®^ Diamond dye which was also detected from BSA samples (Fig. 2c, e) and the lysozyme protein in the user manual. Thus to demonstrate a phosphorylation on the substrate, non-phosphorylation and auto-phosphorylation signals should be removed using corresponding controls.

## Conclusions

We have successfully developed a FDIT method here to improve validations of in vitro protein kinase reactions, the first step before any in vivo analysis to be performed. Taking the high specificity and sensitivity advantages of Pro-Q^®^ Diamond, the FDIT method avoided the disadvantage of high cost in gel staining method through staining samples in a tube with less dye. In this method, a sample only consumes 0.1 ml of Pro-Q^®^ Diamond, which is 1/600 of a typical gel staining cost. Thus, the FDIT method could save up to 95% of the expensive dye consumed in a gel staining method, even if all the 15 sample lanes in each gel were fully loaded. Furthermore, the FDIT method has the advantage of safety over the traditional in vitro kinase reaction using radioactive [γ-^32^P] ATP, and thereafter is a safe and environmental friendly method generating zero radioactive wastes. Moreover, as all the steps in FDIT method were done in one tube before the final quantification in a 96-well plate, it’s easy to multiplex the samples and analyze dozens of samples (96 in our lab) synchronously. The recognition of Pro-Q^®^ Diamond to all phosphorylated proteins broadens its application to any purified proteins but also makes it unsuitable for total plant proteins. Moreover, signals from non-specific binding of the substrate and kinase auto-phosphorylation should be removed using proper controls. As a whole, the FDIT method could serve as a rapid, unambiguous and cost-effective procedure to analyze purified protein phosphorylation in vitro.

## Abbreviations

PTM: post-translational modification
FDIT: fluorescence detection of phosphoprotein in tube
WKS1.1: wheat kinase START 1.1
tAPX: thylakoid ascorbate peroxidase
BSA: bovine serum albumin
OVA: ovalbumin
ASA: ascorbic acid
